# ROS-Mediated Autophagy Induced by Dysregulation of Lipid Metabolism Plays a Protective Role in Colorectal Cancer Cells Treated with Gambogic Acid

**DOI:** 10.1371/journal.pone.0096418

**Published:** 2014-05-08

**Authors:** Haiyuan Zhang, Yunlong Lei, Ping Yuan, Lingjun Li, Chao Luo, Rui Gao, Jun Tian, Zuohua Feng, Edouard C. Nice, Jun Sun

**Affiliations:** 1 Department of Biochemistry and Molecular Biology, Tongji Medical College, Huazhong University of science and Technology, Wuhan, People’s Republic of China; 2 Department of Biochemistry and Molecular Biology, and Molecular Medicine and Cancer Research Center, Chongqing Medical University, Chongqing, China; 3 Monash University, Department of Biochemistry and Molecular Biology, Clayton, Victoria, Australia; University of Manitoba, Canada

## Abstract

Gambogic acid (GA), the main active component of gamboge resin, has potent antitumor activity both *in vivo* and *in vitro*. However, the underlying molecular mechanisms remain unclear. In this study, we found that GA could initiate autophagy in colorectal cancer cells, and inhibition of the autophagy process accelerated the effect of proliferative inhibition and apoptotic cell death induced by GA, implying a protective role of autophagy. Two-dimensional electrophoresis-based proteomics showed that GA treatment altered the expression of multiple proteins involved in redox signaling and lipid metabolism. Functional studies revealed that GA-induced dysregulation of lipid metabolism could activate 5-lipoxygenase (5-LOX), resulting in intracellular ROS accumulation, followed by inhibition of Akt-mTOR signaling and autophagy initiation. Finally, results using a xenograft model suggested ROS-induced autophagy protect against the antitumor effect of GA. Taken together, these data showed new biological activities of GA against colorectal cancer underlying the protective role of ROS-induced autophagy. This study will provide valuable insights for future studies regarding the anticancer mechanisms of GA.

## Introduction

Colorectal cancer (CRC) is the third leading cause of cancer and the fourth for cancer-related deaths worldwide [1], [2]. If CRC can be diagnosed and treated at an early stage, about half of CRC patients could be cured by surgery and multimodal treatment before metastasis occurs [3]. However, to date effective treatment strategies for advanced CRC are limited. 40–50% of patients have metastatic disease, of which 90% die within 5 years of diagnosis [4], [5]. Despite growing advances in molecular medicine, effective early detection, surveillance and treatment of CRC remains a dilemma. Therefore, improved systemic therapeutic strategies are urgently required to effectively eliminate primary or metastatic cancer, for which development of new drugs may be beneficial.

Gambogic acid (GA; C_38_H_44_O_8_, MW 628.76), a polyprenylated xanthone, is a major active ingredient of gamboge isolated from *Garcinia* [6], [7]. It has been reported in Traditional Chinese Medicine that gamboge is cold, acidic, acerbic and poisonous [8]. In Southeast Asia, GA has a long history of use for detoxification, homeostasis, anti-inflammatory and parasiticide medicines [7], [9], [10], [11]. Over the past half-century, pharmacological studies have revealed that GA has strong antitumor activities against various tumors including human leukemia, hepatoma, oral, breast, gastric, pancreatic, prostate, epithelial cervical and lung cancer [9], [12], [13]. Recently, GA has also been reported to have a marked anti-tumor effect for CRC cells *in vivo* and *in vitro* [14], [15]. Due to the wide spectrum of anti-tumor activity with minimal toxicity to normal cells, GA has been approved by the Chinese Food and Drug Administration for the treatment of various cancers and has finished phase II clinical trials [7], [16]. Although GA’s chemical structure was identified in the 1980s from both detailed NMR and X-ray crystallographic studies [12], [17], [18], and multiple antitumor mechanisms (including induction of programmed cell death, cell cycle regulation, telomerase depression, activation of T lymphocytes, angiogenesis inhibition, reactive oxygen species (ROS) generation *etc.*) have been proposed by a number of research groups worldwide, [7], [12], [13], [18], [19], [20], the molecular mechanisms regarding its potent anticancer activity remain ambiguous and require further investigation.

Autophagy is a highly conserved catabolic process characterized by the transport of cellular components from bilayer autophagosomes to lysosomes for degradation and recycling in response to nutrient starvation or metabolic stress [21]. Autophagosome nucleation is initiated by the PI3 kinase type III-Atg6/Beclin 1 complex, while the elongation is monitored by Atg12-Atg5 and Atg8/LC3-phosphatidylethanolamine conjugate systems, both of which are key characteristics of autophagy [21], [22], [23]. Autophagy can be induced by a number of chemotherapeutic agents such as arsenic trioxide and oxaliplatin [24], [25]: however, the role of autophagy in cancer is controversial [26], [27], [28]. A regulated autophagic response can ensure the physiological turnover of damaged organelles and recycled macromolecules to meet the energy demands in response to cytotoxic drugs, leading to prolonged cell survival [26], [29]. By contrast, a massive accumulation of autophagic vacuoles may lead to either autophagic cell death (type II programmed cell death), or an ultimate attempt of the cell to survive depending on tumor type, stage, genetic context and the surrounding cellular environment [26], [29], [30].

Reactive oxygen species (ROS) is a collective term that encompasses incomplete reduction of oxygen, including the superoxide anion (O_2_^-^), hydrogen peroxide (H_2_O_2_) and the hydroxyl radical (HO•) [31], [32]. Major sources of cellular ROS are generated from the mitochondrial electron transport chain (Mito-ETC), the NADPH oxidase (NOX) complex and the endoplasmic reticulum [31], [32]. Cancer cells from advanced stage tumors frequently exhibit high oxidative stress, suggesting that increased levels of ROS play an important role in tumor progression and also engender cancer cells with a lower tolerance for ROS [33], [34]. Activation of oncogenes, loss of functional p53, aberrant metabolism and chemical treatment have been reported to increase ROS production in cancer cells [33], [34]. The intracellular redox homeostasis is a key determinant of cell fate: excessive production of ROS usually results in cytotoxic effects and may lead to apoptotic cell death, while moderate levels of ROS can act as a second-messenger for regulation of diverse cellular processes such as cell survival, proliferation and metastasis [35], [36], [37]. Accumulation of ROS has been reported to associate with the initiation of autophagy and be invariably involved in the outcome of autophagy (cell survival or death) [38], [39]. It is generally accepted that ROS can induce autophagy, and that autophagy, in turn, assists in the clearance of excessive ROS to protect cells from oxidative damage, which may reflect the balance of either cell survival or death [28], [38], [39].

Recent studies show that GA can induce the accumulation of ROS in cancer cells contributing to the anti-cancer activity [40], [41], [42], while autophagy can inhibit the therapeutic effect of GA on glioblastoma cells [43]. In this study, we found that GA could promote apoptosis and autophagy in colorectal cancer cells *in vitro* and *in vivo*, and inhibition of autophagy enhanced the sensitivity against GA treatment. In addition, the accumulation of intracellular ROS arising from 5-LOX was required for GA-induced autophagy.

## Materials and Methods

### Cell Culture and Reagents

Human colon carcinoma cell lines, HCT116 and SW620, and the murine colon carcinoma cell line C26 were purchased from ATCC. Cells were cultured in DMEM supplemented with 10% fetal bovine serum, 10^5 ^U/L penicillin and 100 mg/L streptomycin at 37°C in an atmosphere containing 5% CO_2_.

The following reagents were used in this study: Gambogic acid (Gaia Chemical Corp, G1000), MTT (Sigma, M2128), 3-methyladenine (3-MA) (Sigma, M9281), NAC (Sigma, A9165), Z-VAD-fmk (Sigma, V116), acridine orange (Sigma, A6014), Dimethyl Sulfoxide (DMSO) (Sigma, D2650) Apocynin (Sigma, A10809), Rotenone (Sigma, R8875), Nordihydroguaiaretic Acid (NDGA) (Sigma, 74540), Pepstatin A (Sigma, P4265), E64d (Sigma, E8640). For storage, a 10 mM solution of GA was prepared in DMSO, stored at −20°C, and then diluted as needed in culture medium.

Antibodies against the following proteins were used: Cleaved Caspase 3 (Cell signaling, 9664S), Beclin 1 (Santa Cruz, sc-11427), LC3 (Abcam, ab58610), Atg5 (Abcam, ab78073), Atg7 (Abcam, ab53255), p62 (Abcam, ab91526), 5-LOX (Abcam, ab39347), actin (Santa Cruz, sc-1616), Akt (Cell signaling, 4685), phosphor-Akt (Cell signaling, 4051), mTOR (Cell signaling, 2983), phosphor-mTOR (Cell signaling, 2971), p70 S6K (Santa Cruz, sc-9027), phosphor-p70 S6K (Santa Cruz, 7984-R), horseradish peroxidase (HRP)-conjugated anti-rabbit secondary antibody (Santa Cruz, sc-2004), HRP-conjugated anti-mouse secondary antibody (Santa Cruz, sc-2005).

### Cell Viability Assay

Cells were seeded in 96-well culture plates and treated for 12 h, 24 h and 36 h respectively. Subsequently cell viability was evaluated using the MTT assay [44]. Absorbance was measured at 490 nm (test wavelength) and 570 nm (reference wavelength) with a multi-well spectrophotometer (MDC, Sunnyvale, CA).

### Annexin V-FITC/PI Double-labeled Flow Cytometry

To detect the apoptotic ratio of cells treated with GA (0.25, 0.5 or 1.0 µM), the expression of Annexin V-FITC and the exclusion of PI were detected using two-color flow cytometry (FCM). HCT116 or SW620 cells were collected using EP tubes, washed twice with PBS and resuspended in 500 µl binding buffer. The samples were incubated with 5 µL Annexin V-FITC for 10 min at room temperature and then 5 µL PI was added. Each sample was incubated for a further 10 min at room temperature in the dark before the fluorescence intensity was quantitated using a flow cytometer (Beckman Coulter, Miami, FL, USA).

### GFP-LC3 Staining of Autophagosomes

HCT116 and SW620 cells were transfected with a pEGFP-LC3 plasmid (referred to as GFP-LC3) using lipofectamine 2000 (Invitrogen, 11668027) according to the manufacturer’s instructions. The fluorescence of GFP-LC3 was viewed and the rate of GFP-LC3-labled vacuole formation (autophagosomes) was counted under a fluorescence microscope [45], [46]. Cells with GFP-LC3 punctate dots were defined as positive if cells that had 5 or more GFP-LC3 dots in the cytoplasm [47].

### Detection of Acidic Vesicular Organelles

Cells (1×10^5^) were plated in 6-well plates. Following drug treatment, cells were stained with 1 µg/mL acridine orange for 15 min, washed with PBS and examined by fluorescence microscopy [48], [49].

### Electron Microscopy

Cells were harvested, pelleted and fixed in paraformaldehyde (0.1% glutaraldehyde in 0.1 M sodium cacodylate) for 2 h, postfixed with 1% OsO_4_ for 1.5 h, washed and finally stained for 1 h in 3% aqueous uranyl acetate. The samples were then rinsed with water again, dehydrated with graded alcohol (50%, 75% and 95–100% alcohol) and embedded in Epon-Araldite resin (Canemco, 034). Ultrathin sections were cut on a Reichert Ultramicrotome, counterstained with 0.3% lead citrate and examined on a Philips EM420 transmission electron microscope. Cells with autophagic vacuoles were defined as positive if they had 5 or more autophagic vacuoles. The area occupied by autophagic vacuoles and the cytoplasm were determined with Image Pro Plus Image Analysis Software version 3 and used to calculate the cytoplasmic area occupied by the autophagic vacuoles [50].

### TUNEL Assay

TUNEL assay was performed using the DeadEnd Fluorometric TUNEL system (Promega, G3250) according to the manufacturer’s instructions. TUNEL positive cells were examined under a fluorescence microscope [51].

### RNA Interference

*Atg5*, *Beclin 1, 5-LOX* and negative control siRNA were synthesized by Genepharma. The sequences of siRNA were as following: human *Atg5* siRNA, sense 5′-GAC GUU GGU AAC UGA CAA ATT-3′ and antisense 5′-UUU GUC AGU UAC CAA CGU CTT-3′; human *Beclin 1*siRNA, sense 5′-GGA GCC AUU UAU UGA AAC UTT-3′ and antisense 5′-AGU UUC AAU AAA UGG CUC CTT-3′. 5-LOX was designed according to previous study (targeting sequence: 5′-GCGCAAGTACTGGCTGAATGA-3′; NM_000698) [52]. The siRNA were transfected with Lipofectamine 2000 reagent (Invitrogen, 11668027) for 24 h in HCT116 cells according to the manufacturer’s protocol.

### Reactive Oxygen Species (ROS) Measurement

Intracellular ROS level was detected by staining cells with 2′, 7′-dichlorofluorescein diacetate (DCFH-DA) (GENMED, GMS10016.2) according to the manufacturer’s instructions. The DCFH-DA signal was measured with a Molecular Devices SPECTRAMAX M5 fluorimeter (490 nm excitation and 530 nm emission).

### Immunoblot

Proteins were extracted in RIPA buffer (50 mM Tris-base, 1.0 mM EDTA, 150 mM Nacl, 0.1% SDS, 1% TritonX-100, 1% Sodium deoxycholate, 1 mM PMSF) and quantified with the DC protein assay kit (Bio-Rad). Samples were separated by 12% SDS-PAGE and transferred to PVDF membranes. The membranes were blocked overnight with Tris-buffered saline with Tween 20 (TBST) (20 mM Tris-HCl, 150 mM NaCl, 0.1% Tween 20) in 5% skimmed milk at 4°C, and subsequently probed using the primary antibodies: rabbit-anti-Beclin 1 (diluted 1∶500), rabbit-anti-Atg5 (diluted 1∶1,000), rabbit-anti-atg7 (diluted 1∶500), rabbit-anti-LC3 (diluted 1∶1,000), rabbit-anti-5-LOX (diluted 1∶1,000), rabbit-anti-Akt (diluted 1∶1,000), rabbit-anti-phospho-Akt (diluted 1∶1,000), rabbit-anti-mTOR (diluted 1∶1,000), rabbit-anti-phospho-mTOR (diluted 1∶1,000). Blots were incubated with the respective primary antibodies for 2 h at room temperature. After washing three times in TBST, the blots were incubated with HRP-conjugated anti-rabbit secondary antibody (diluted 1∶5,000) or HRP-conjugated anti-mouse secondary antibody (diluted 1∶6,000) for 1 h at room temperature. Blots were visualized using immobilon western chemiluminescence reagents (Millipore, WBKLS0500).

As a measure of autophagic flux, immunoblots for LC3 were performed in the absence or presence of lysosomal enzyme inhibitors. LC3 flux was determined by the ratio of densitometric value of LC3-II relative to the corresponding DMSO-treated control without drug treatment as described elsewhere [23], [53].

### 2-DE and MS/MS Analysis

2-DE and MS/MS analysis was performed as described previously [54]. Briefly, cells were dissolved in lysis buffer (7 M urea, 2 M thiourea, 4% CHAPS, 100 mM DTT, 0.2% pH3–10 ampholyte, Bio-Rad, USA) in presence of protease inhibitor (Sigma). Samples were loaded into IPG strips (17 cm, pH3–10NL, Bio-Rad) using a passive rehydration method, and then subjected to isoelectric focusing (Bio-Rad). The second dimension separation was performed using 12% SDS-PAGE after equilibration. The gels were stained with CBB R-250 (Bio-Rad). Identification and quantitation of protein spots in the gel was achieved using PDQuest software (Bio-Rad).

In-gel protein digestion was performed using mass spectrometry grade trypsin according to the manufacturer’s instructions. The gel spots were destained with 100 mM NH_4_HCO_3_/50% acetonitrile (ACN) and dehydrated with 100% ACN. The gels were then incubated with trypsin (Promega, V5280), followed by double extraction with 50% ACN/5% trifluoroacetic acid (TFA). The peptide extracts were dried in a speed-VAC concentrator (Thermo), and subjected to mass spectrometric analysis using a Q-TOF mass spectrometer (Micromass, Manchester, UK) fitted with an ESI source.

### Immunohistochemistry

Immunohistochemistry was performed using the Dako EnVision System (Dako Cytomation GmbH, Hamburg, Germany). Consecutive paraffin wax-embedded tissue sections (3–5 µm) were dewaxed and rehydrated. Antigen retrieval was performed by pretreatment of the slides in citrate buffer (PH 6.0) in a microwave oven for 12 min. Thereafter slides were cooled to room temperature in deionized water. Endogenous peroxidase activity was quenched by incubating the slides in methanol containing 3% hydrogen peroxide followed by washing in PBS for 5 min after which the sections were incubated for 1 h at room temperature with normal goat serum and subsequently incubated at 4°C overnight with the primary antibodies. Next the sections were rinsed with washing buffer (PBS with 0.1% bovine serum albumin) and incubated with horseradish peroxidase-linked goat anti-rabbit antibodies followed by reaction with diaminobenzidine and counterstaining with Mayer’s hematoxylin.

### Tumor Xenograft Model

Experimental protocols were carried out in compliance with the European Communities Council Directive of 24 November 1986 (86/609/EEC) with the approval of the Ethics Committee of Tongji Medical College. Healthy female mice (BALB/c, 6–8 weeks of age, non-fertile and 18–20 g each) were injected subcutaneously with C26 cells (one million cells per mouse). When tumors were approximately 5 mm×5 mm in size (usually ten days after inoculation), the animals were randomly pair-matched into two groups (nine mice per group) as follows: a control group (intraperitoneal injection of vehicle: 5% DMSO, 50% PEG-400 in PBS) and a gambogic acid group (intraperitoneal injection of 8 mg/kg gambogic acid once every other day for six times). The tumor volumes were evaluated as follows: tumor volume (mm^3^) = (length×width^2^)/2. Animals were sacrificed 12 days after injection. Tumors were dissected and frozen in liquid nitrogen or fixed in formalin immediately.

To test the efficacy of combinative treatments, when tumors were approximately 600 mm^3^, the animals were pair-matched into four groups (8 mice per group): a control group, GA, GA+NAC, and GA+3-MA. Control group: intraperitoneal injection of vehicle: 5% DMSO, 50% PEG-400 in PBS. GA treatment: intraperitoneal injection of 8 mg/kg gambogic acid once every other day for ten times. NAC treatment: animals received either deionized water or water containing NAC (7 mg/mL; neutralized to pH 7.4 with NaOH); We assumed an average mouse weight of 25 g and daily water consumption of 6.7 mL, the estimated daily maternal dose was 1.9 g/kg/d [55]. 3-MA treatment: subcutaneous injections of saline (control) or 1 mg/kg 3-MA, and the injection were repeated every day [56]. The tumor volumes were evaluated as follows: tumor volume (mm^3^) = (length×width^2^)/2.

### Statistical Data Analysis

Comparisons between two groups were performed by Student’s test. Statistical significance was defined as ^*^p<0.05; ^**^p<0.01;^ ***^p<0.001.

## Results

### GA Induces Apoptosis in Colorectal Cancer Cells

**Figure 1:**
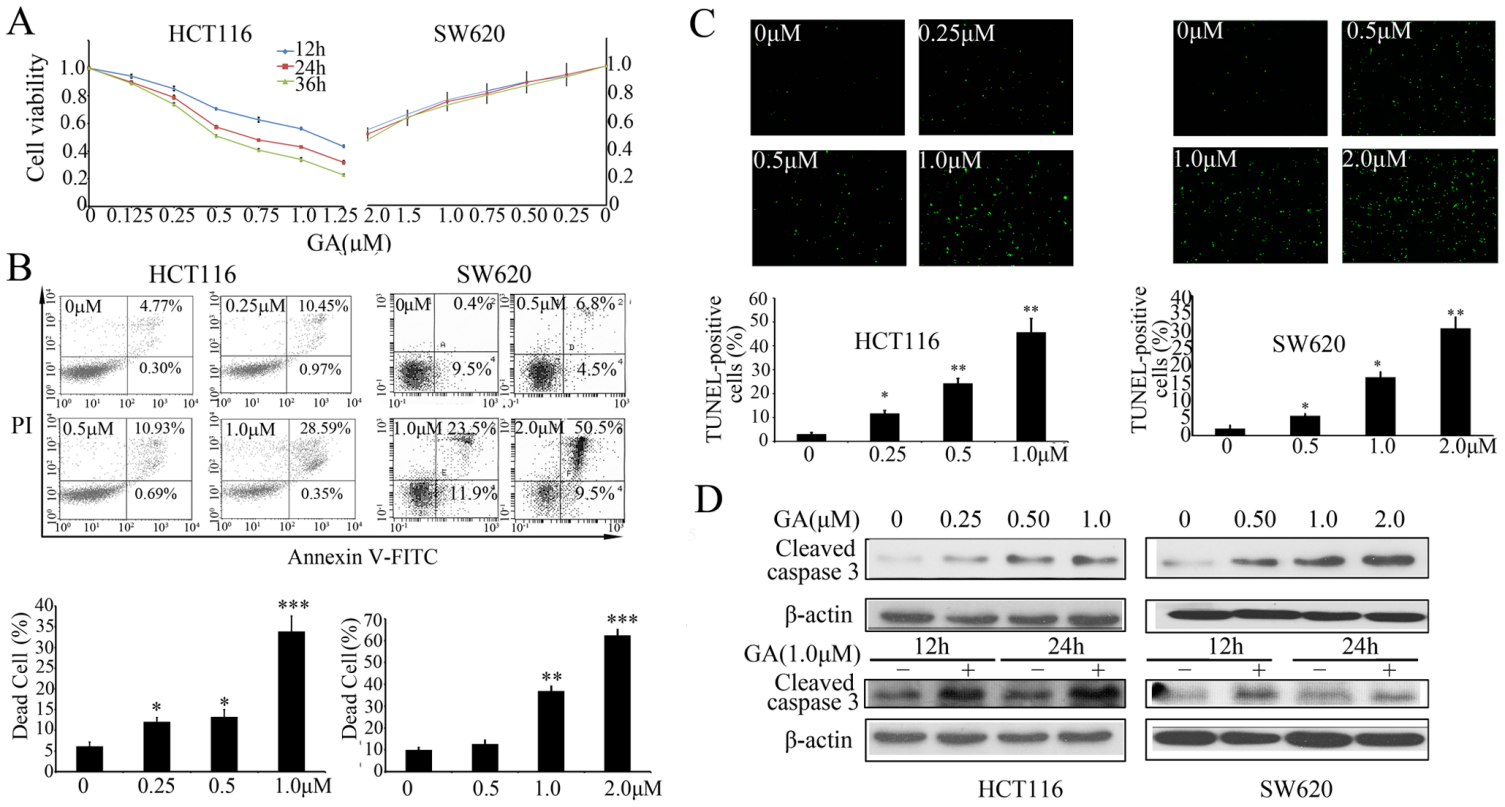
GA promotes apoptosis in colorectal cancer cells. (**A**) HCT116 and SW620 cells were treated with increasing concentrations of GA for 12 h, 24 h or 36 h, and the cell viability index was measured by MTT assay. (**B**) HCT116 and SW620 cells were treated with increasing concentrations of GA for 24 h, cell apoptosis was detected by annexin-V fluorescein isothiocyanate (FITC) and propidium iodide (PI) double staining followed by flow cytometry analysis. Dot plot display of Annexin-V FITC-fluorescence versus propidium iodide fluorescence is shown in logarithmic scale. Living cells tested negative for both annexin V-FITC and PI. Populations testing annexin V positive/PI negative were classified as early-stage apoptotic cells, and double-positive cells were classified as dead cells. Bar diagram showing the percentage of dead cells after different treatments. (**C**) HCT116 and SW620 cells were treated with increasing concentrations of GA for 24 h, dead cells were detected by TUNEL assay. The TUNEL-positive cells were counted from at least 100 random fields. (**D**) Immunoblot analysis of cleaved-caspase 3 from lysates of HCT116 and SW620 cells treated with various concentrations of GA for 24 h, or treated with 1 µM GA for 12 h and 24 h.

To determine the effect of GA on colorectal cancer cells, HCT116 and SW620 cells were treated with different concentrations of GA for 12 h, 24 h or 36 h, respectively. MTT assay was used to determine cell viability. As shown in Figure 1A, treatment with GA resulted in proliferative inhibition of HCT116 cells in both a dose- and time- dependent manner with IC_50_ values of about 1.1 µM, 0.6 µM and 0.5 µM for 12 h, 24 h and 36 h, respectively. SW620 cells showed only dose-dependent inhibition with an IC_50_ value of about 2 µM. In addition, the effect of GA on colorectal cancer cell death was examined using Annexin-V fluorescein isothiocyanate (FITC) and propidium iodide (PI) double staining, as well as TUNEL assays. As shown in Figure 1B, the percentage of Annexin-V positive cells, which is indicative of dead cells, was significantly increased after treatment with GA. These results were consistent with those obtained from the TUNEL assay (Figure 1C). Next, we examined whether GA-induced cell death was caspase-dependent. As shown in Figure 1D, cleaved-caspase 3 was accumulated upon GA treatment. Moreover, GA induced cell death could be markedly reversed by a pan-caspase inhibitor, Z-VAD-fmk (Figure S1), suggesting that GA induced a caspase-dependent apoptotic cell death.

### GA Initiates Autophagy in Colorectal Cancer Cells

**Figure 2:**
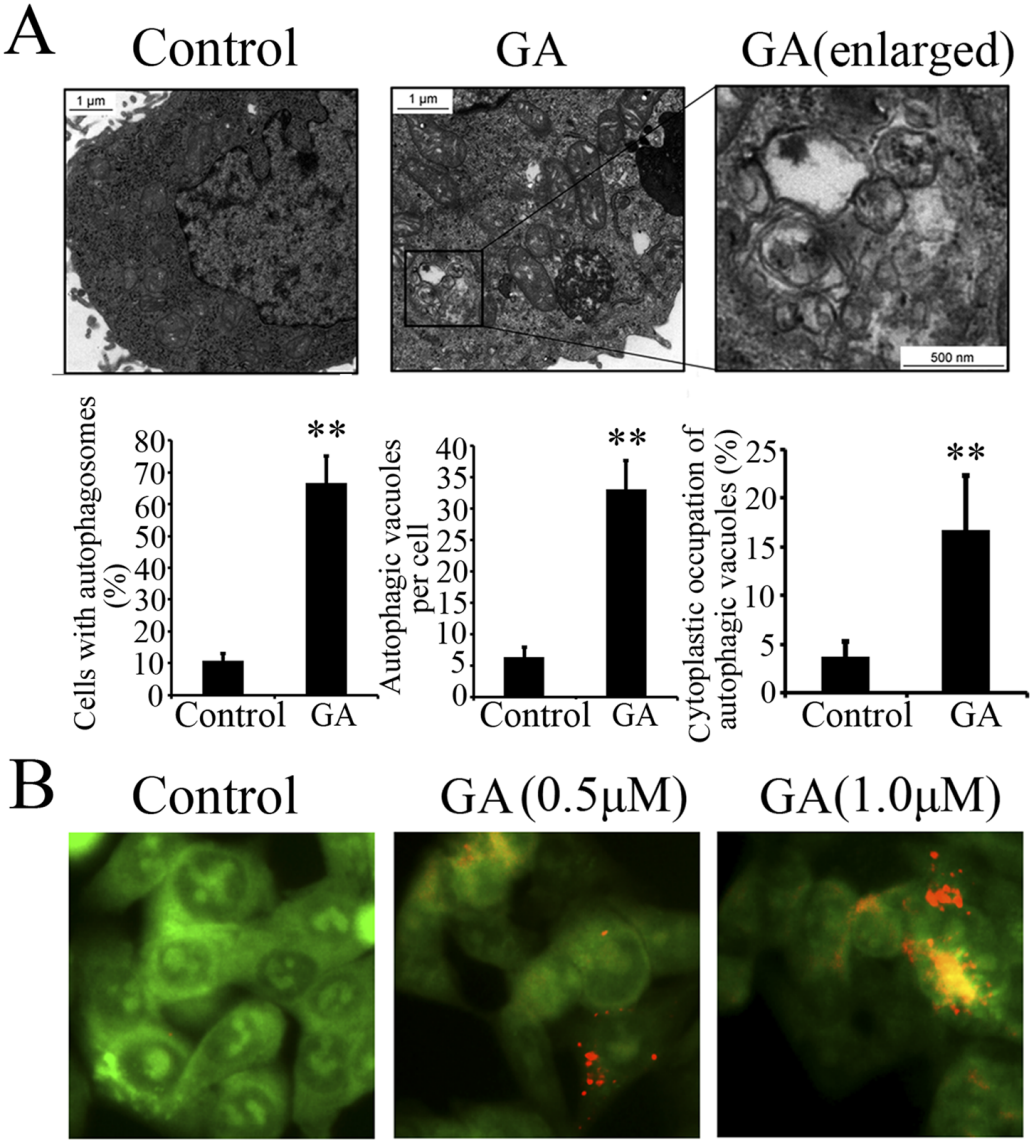
GA induced formation of autophagic vacuoles and AVOs in colorectal cancer cells. (**A**) Upper Panels. Representative transmission electron micrographs depicting ultrastructures of HCT116 cells treated with either DMSO (control, <0.1%) or 1 µM GA for 24 h. Lower panels. The cells with autophagic vacuoles were defined as cells that had five or more autophagic vacuoles. The percentage of the cells with autophagosomes and the average number of vacuoles per cell were analyzed from at least 100 randomly chosen TEM fields. Scale bars: 1 µm; 100 nm (indicated enlargements). (**B**) Acridine orange staining in HCT116 cells treated with DMSO (control, <0.1%), 0.5 µM GA or 1.0 µM GA for 12 h. All data are representative of three independent experiments. ** p<0.01.

To better understand the anti-cancer effect of GA, the ultrastructure of HCT116 cells treated with GA or DMSO (<0.1%) was analyzed by transmission electron microscopy (TEM). Numerous membrane-bound vacuoles, characteristic of autophagosomes, were observed in the cytoplasm of GA-treated cells, whereas membrane-bound vacuoles could rarely be found in the cells treated with DMSO (Figure 2A). In addition, acridine orange staining was used to analyze the formation of acidic vesicular organelles (AVOs), another major feature of autophagy. As shown in Figure 2B, HCT116 cells treated with GA resulted in obvious formation of yellow-orange AVOs compared with the DMSO-treated cells.

**Figure 3:**
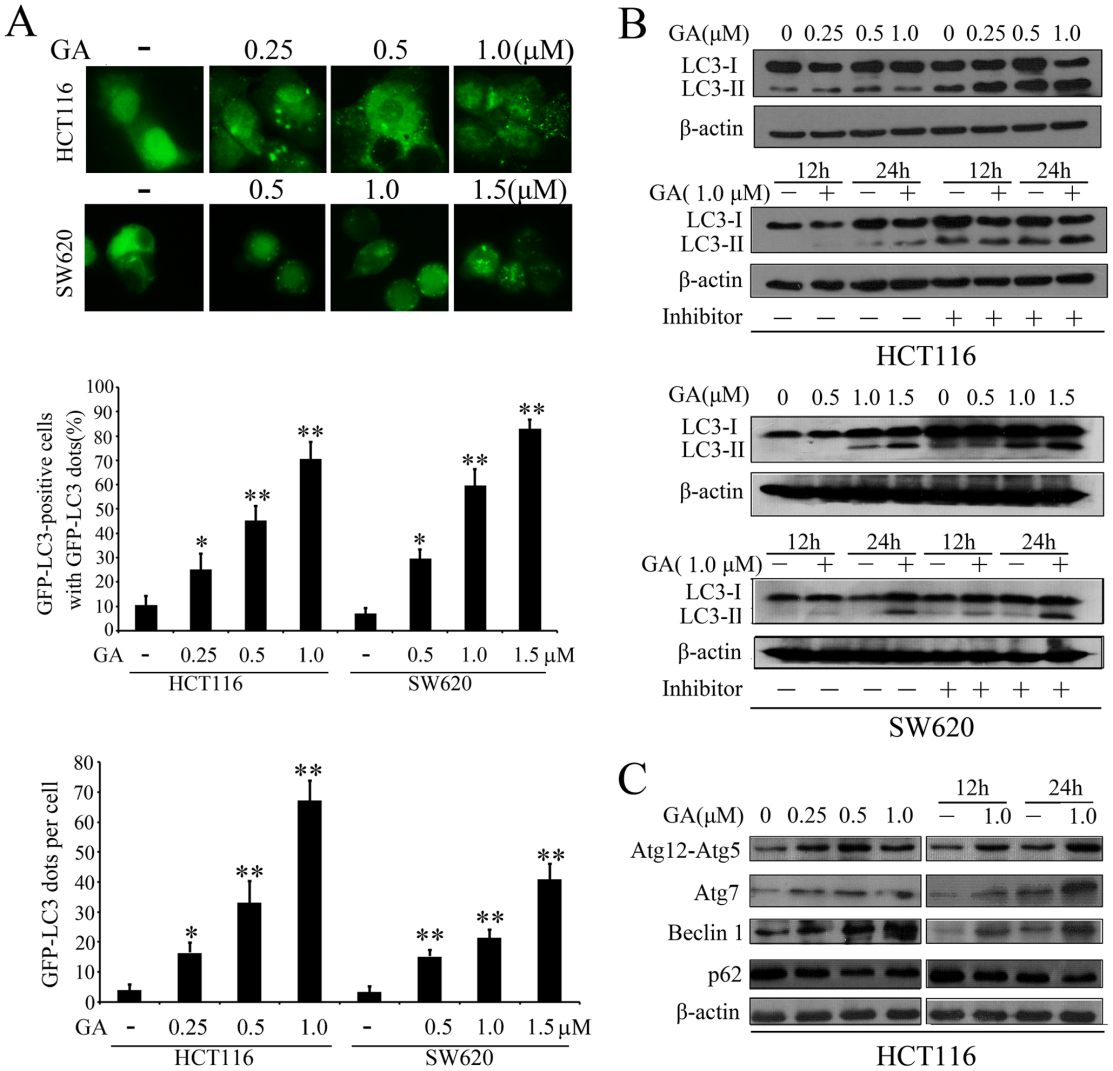
GA initiates autophagy in colorectal cancer cells. (**A**) HCT116 and SW620 cells transfected with a pEGFP-LC3 plasmid were treated with indicated concentrations of GA for 24 h. Cells were defined as positive if they had 5 or more GFP-LC3 dots in the cytoplasm. The percentage of the cells with GFP-LC3 dots and the average number of GFP-LC3 dots per cell were analyzed from at least 100 random fields. (**B**) Immunoblot analysis of the conversion of LC3-I to LC3-II in HCT116 cells and SW620 cells after treated with indicated concentrations of GA for 24 h, or with 1.0 µM of GA for 12 h and 24 h in the absence or presence of lysosomal inhibitors (E64d and pepstatin each at 10 µg/ml). (**C**) Immunoblot analysis of the expression level of Atg12-Atg5 conjugate, Atg7, Beclin 1 and p62 after treated with indicated concentrations of GA for 24 h, or with 1 µM of GA for 12 h and 24 h. Actin served as a loading control. * p<0.05; ** p<0.01.

The localization and aggregation of LC3 is known to be important for transport and maturation of the autophagosome [57]. Therefore, pEGFP-LC3 plasmid was transiently transfected into both HCT116 and SW620 cells to further confirm whether GA initiates autophagy in colorectal cancer cells. As indicated in Figure 3A, the percentage of GFP-LC3-positive cells and average amount of GFP-LC3 dots were both significant increased upon GA treatment in a dose-dependent manner. The lipidated form of LC3 transforming from LC3-I to LC3-II is correlated with the extent of autophagosome formation [57]. GA also markedly enhanced the turnover from LC3-I to LC3-II, which was further accumulated in the presence of E64d and pepstatin A (both lysosomal protease inhibitors) (Figure 3B), suggesting that GA could enhance autophagic flux. In addition to LC3, the expressions of a series of autophagic related proteins, including p62, Beclin 1, Atg7 and Atg12-Atg5, have been shown to be altered during autophagy [23]. Therefore, we investigated the expression of these proteins upon GA treatment. As shown in Figure 3C, GA upregulated the expression of Beclin 1, Atg7 and Atg12-Atg5 in a dose-dependent manner, while the accumulation of p62 was decreased. These results further demonstrated that GA can induce the formation of autophagosomes in colorectal cancer cells.

### Blockage of Autophagy Enhances GA-induced Apoptosis

**Figure 4:**
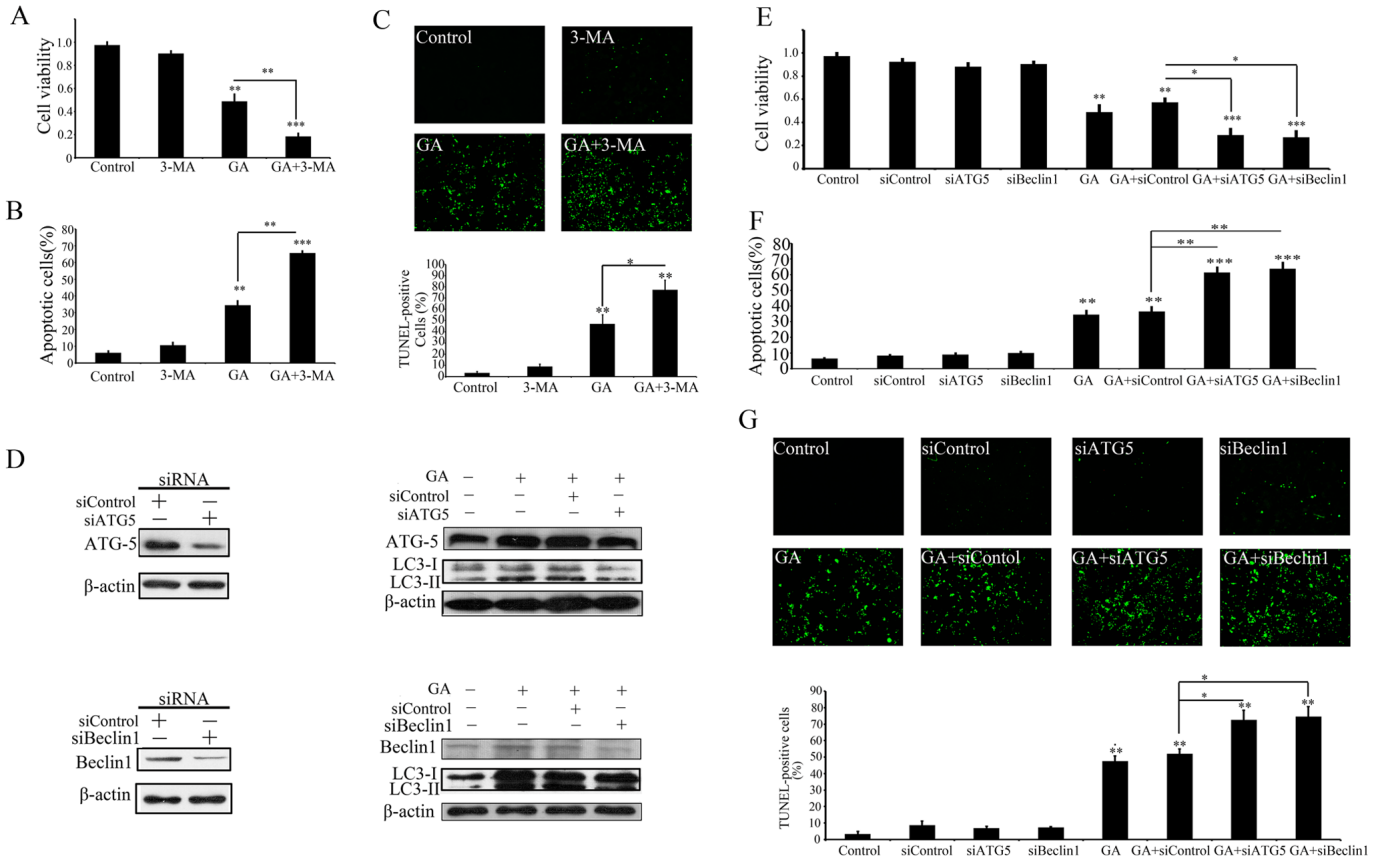
Blockage of autophagy enhances GA-induced apoptosis. (**A–C**) HCT116 cells were treated with vehicle control (1‰ DMSO, Control), 3-MA, 1 µM GA (GA), or 1 µM GA in the presence 3-MA (GA+3-MA) for 24 h. And then the cell viability was evaluated by MTT assay (A), and the apoptotic effect was detected by PI/annexin-V staining (B) and TUNEL assays(C). (**D**) Immunoblot detection of the expression of ATG5, beclin-1 and LC3 in HCT116 cells treated with GA in the present or absent with siATG5 or siBeclin 1. (**E–G**) HCT116 cells were treated with Lipofectamine 2000 (Control), control siRNA (siControl), siATG5 (siATG5), 1 µM GA (GA), GA in the presence control siRNA (GA+siControl), siATG5 (GA+siATG5) or siBeclin1 (GA+siBeclin 1) for 24 h. And then the cell viability was evaluated by MTT assay (E), and the apoptotic effect was detected by PI/annexin-V staining (F) and TUNEL assays (G). * p<0.05; ** p<0.01.

Considering the paradoxical role of autophagy in promoting cell death or survival, we further treated colorectal cancer cells with a commonly used autophagy inhibitor (3-MA) either alone or in combination with GA to determine the functional role of autophagy in GA-induced apoptosis. As shown in Figure 4A, pre-treatment of HCT116 cells with 3-MA significantly enhanced the effect of GA-induced proliferative suppression. Consistent with this, the results of Annexin-V/PI double staining (Figure 4B) and TUNEL assays (Figure 4C) also showed that GA in combination with 3-MA exhibited a stronger pro-apoptotic effect compared with GA alone. In addition, transient transfection with Atg5- or beclin1-targeted siRNA to ablate Atg5 or beclin 1 expression can inhibit GA-induced LC3-II accumulation, as well as augment the anti-proliferative and pro-apoptotic effects of GA in HCT116 cells (Figure 4D–G). These data suggest that autophagy protects GA-treated colorectal cancer cells from apoptotic cell death.

### Redox Dysregulation was Induced upon GA Treatment

**Figure 5:**
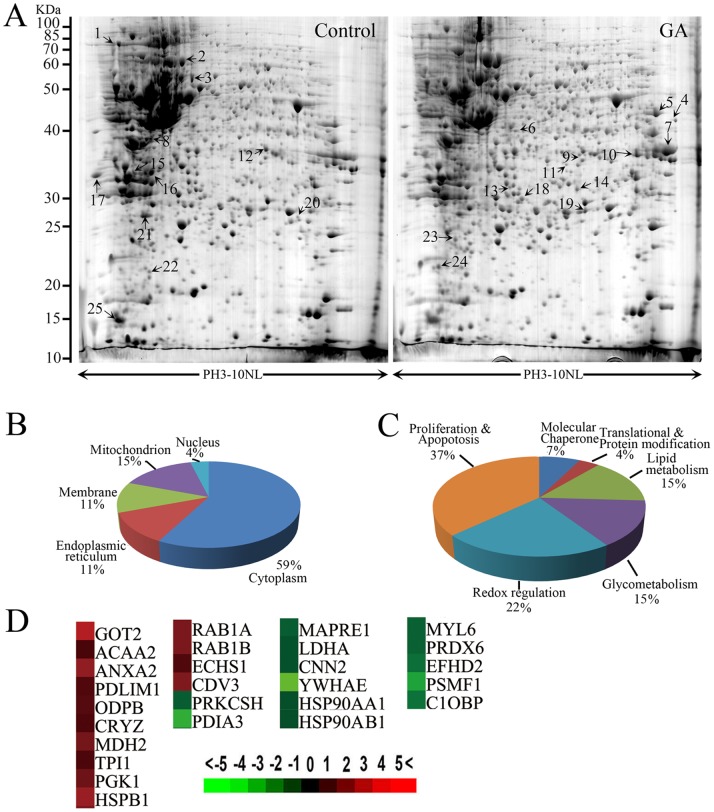
Redox dysregulation was induced upon GA treatment. (**A**) Representative two-dimensional gel images of the control and GA-treated (1 µM, 24 h) HCT116 cells. Total protein extracts were separated on pH 3–10 nonlinear immobilized pH gradient strips in the first dimension followed by 12% SDS-PAGE in the second dimension and visualized by CBB staining. (**B**) The identified proteins were categorized into groups according to their subcellular locations. (**C**) 27 distinct proteins were classified into 6 groups based on their biological functions. (**D**) Protein cluster map generated by cluster software. Expression of proteins in the control was constant at 0, whereas proteins upregulated in GA-treated cells are in red, and the downregulated proteins are in green. The intensity of the color green or red corresponds to the degree of alteration, respectively, according to the color strip at the bottom of the figure.

To explore the mechanism by which GA induces autophagy, we profiled differentially expressed proteins in HCT116 cells treated with or without GA. By comparing 2-DE patterns, differentially expressed proteins were defined as statistically meaningful (p<0.05) if both following two criteria were met: 1) intensity alterations of >2.0-fold and 2) observed in at least three individual experiments. 25 spots that met these criteria were selected and analyzed using ESI-Q-TOF tandem mass spectrometry, and a total of 27 proteins were identified (Fig. 5A, Table I). The MS/MS data were queried using the search algorithm MASCOT against the Expasy protein sequence database. Proteins were identified based on a number of criteria including pI, MW, peptide identification, and coverage (Table I). Of these, 13 proteins were down-regulated whereas 14 proteins were up-regulated post GA treatment (Fig. 5D). The identified proteins were divided into various groups based on their subcellular localization and biological functions (Fig. 5B and 5C). The proteins were found to be located in the cytoplasm (59%), nucleus (4%), mitochondrion (15%), cell membrane (11%), or endoplasmic reticulum (11%). This implicated roles in Proliferation and Apopotosis (37%), Redox regulation (22%), Lipid metabolism (15%), Glycometabolism (15%), Translational & Protein modification (4%), and Molecular Chaperone (7%). Following Proliferation and Apopotosis, the next most altered proteins were involved in redox regulation upon GA treatment, suggesting ROS may be involved in GA-induced autophagy.

**Table 1:** Protein spots identified by ESI-Q-TOF.

Spot No.	Accession No.	Protein Name	Gene Name	Theoretical Mr	Theoretical pI	Peptide (95%)	Coverage (95%)
1	P08238	Heat shock protein HSP 90-beta	HSP90AB1	83133	4.96	42	41.8
	P07900	Heat shock protein HSP 90-alpha	HSP90AA1	84528	4.94	37	35.2
2	P14314	Glucosidase 2 subunit beta	PRKCSH	57816	4.33	48	46.7
3	P30101	Protein disulfide-isomerase A3	PDIA3	54265	5.61	103	62.3
4	P00505	Aspartate aminotransferase	GOT2	44737	8.98	46	57.2
5	P00558	Phosphoglycerate kinase 1	PGK1	44483	8.30	217	86.5
6	P42765	3-ketoacyl-CoA thiolase, mitochondrial	ACAA2	41924	8.32	5	16.6
7	P07355	Annexin A2	ANXA2	38472	7.56	92	76.3
8	P00338	L-lactate dehydrogenase A chain	LDHA	36557	8.46	17	43.9
9	O00151	PDZ and LIM domain protein 1	PDLIM1	35940	6.55	4	17.3
10	P11177	Pyruvate dehydrogenase E1 component subunit beta	ODPB	35904	5.38	12	18.9
11	Q08257	Quinone oxidoreductase	CRYZ	35075	8.57	4	12.2
12	Q99439	Calponin-2	CNN2	33565	6.92	38	66.0
13	P40926	Malate dehydrogenase	MDH2	33000	8.54	81	70.1
14	P60174	Triosephosphate isomerase	TPI1	30791	5.65	49	62.2
15	Q15691	Microtubule-associated protein RP/EB family member 1	MAPRE1	29867	5.02	47	67.5
16	Q92530	Proteasome inhibitor PI31 subunit	PSMF1	29816	5.42	20	54.6
17	P62258	14-3-3 protein epsilon	YWHAE	29173	4.63	25	58.8
18	P30084	Enoyl-CoA hydratase, mitochondrial	ECHS1	28342	5.88	11	34.1
19	Q9UKY7	Protein CDV3 homolog	CDV3	27203	6.08	12	66.6
20	Q96C19	EF-hand domain-containing protein D2	EFHD2	26566	5.15	20	41.6
21	P30041	Peroxiredoxin-6	PRDX6	24903	6.02	2	9.3
22	Q07021	Complement component 1 Q subcomponent-binding protein, mitochondrial	C1QBP	23783	4.32	1	4.6
23	P04792	Heat shock protein beta-1 cell death	HSPB1	22782	5.98	52	74.1
24	P62820	Ras-related protein Rab-1A	RAB1A	22546	5.93	10	26.3
	Q9H0U4	Ras-related protein Rab-1B	RAB1B	22017	5.25	7	27.3
25	P60660	Myosin light polypeptide 6	MYL6	16798	4.56	47	64.9

### ROS is Required for GA-induced Autophagy

**Figure 6:**
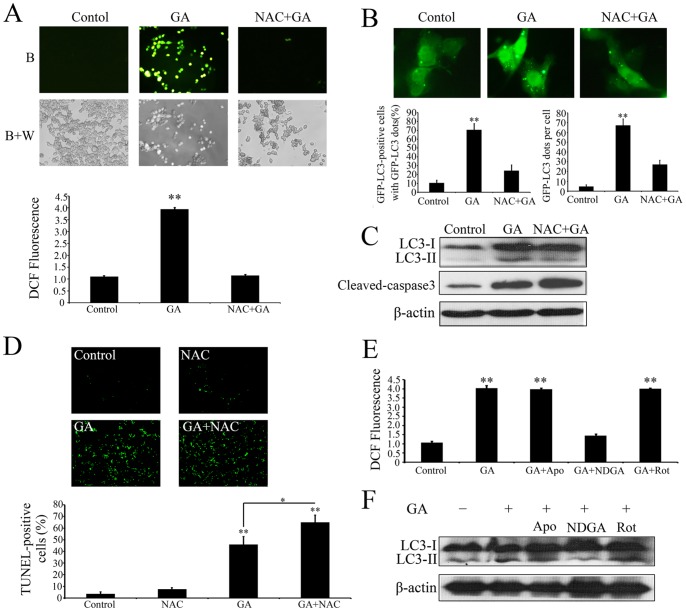
ROS is required for GA-induced autophagy. (**A**) Intracellular ROS in HCT116 cells treated with 1‰ DMSO (Control), 1.0 µM GA (GA), or 1.0 µM GA in the presence NAC (10 mM) (GA+NAC) for 24 h were detected by staining cells with 2′,7′-dichlorofluorescein diacetate under blue (B), or blue and white excitation (B+A). The DCFH-DA signal was measured using a Molecular Devices SPECTRAMAX M5 fluorimeter. (**B**) HCT116 cells transfected with a pEGFP-LC3 plasmid with or without GA (1.0 µM) or/and NAC (10 mM) for 24 h. Cells were defined as positive if they had 5 or more GFP-LC3 dots in the cytoplasm. The percentage of the cells with GFP-LC3 dots and the average number of GFP-LC3 dots per cell were analyzed from at least 100 random fields. ** p<0.01. (**C**) Immunoblot analysis of the conversion of LC3-I to LC3-II and expression of cleaved-caspase 3. (**D**) HCT116 cells treated with or without GA (1.0 µM) or/and NAC (10 mM) for 24 h, cell apoptosis was detected by TUNEL assay. The TUNEL-positive cells were counted from at least 100 random fields. (**E**) HCT116 cells were treated with or without GA or/and the antioxidants Apocynin, rotenone and nordihydroguaiaretic acid (NDGA) for 24 h, and then the intracellular ROS were measured using a Molecular Devices SPECTRAMAX M5 fluorimeter. (**F**) HCT116 cells were treated with or without GA or/and the antioxidants Apocynin, rotenone and nordihydroguaiaretic acid (NDGA) for 24 h, and then the conversion of LC3-I to LC3-II were detected by immunoblot analysis.

To determine whether ROS was involved in GA-induced autophagy, we firstly examined levels of intracellular ROS of colorectal cancer cells treated with or without GA. As shown as Figure 6A, GA triggered massive ROS accumulation in HCT116 cells. To evaluate the potential significance of ROS in GA-induced autophagy and apoptosis, N-acetyl-cysteine (NAC), a general ROS inhibitor, was applied to block intracellular ROS generation. The results demonstrated that NAC treatment could completely reverse GA-induced ROS production (Figure 6A), which markedly attenuated GA-induced formation of GFP-LC3 dots (Figure 6B) and conversion of LC3-I to LC3-II (Figure 6C), while enhanced GA-induced accumulation of cleaved-caspase 3 (Figure 6C) and cell death (Figure 6D), suggesting ROS potentiate GA-induced autophagic processes and inhibit the antitumor effect of GA.

Major sources of cellular reactive oxygen species (ROS) are generated from the mitochondrial electron transport chain (Mito-ETC) and the NADPH oxidase (NOX) complex [58], [59]. In addition, our proteomics data showed that multiple proteins that were altered upon GA treatment (about 15% total altered proteins) were involved in lipid metabolism (Figure 5C), dysregulation of which could promote ROS accumulation through 5-LOX. To clarify the source of GA-induced ROS, the antioxidants apocynin, rotenone and nordihydroguaiaretic acid (NDGA) were used to block NADPH oxidase, mitochondrial and 5-LOX-driven ROS release, respectively. As shown as Figure 6E, only treatment with NDGA reduced the level of GA-induced intracellular ROS, and inhibited GA-induced conversion of LC3-I to LC3-II (Figure 6F), suggesting GA-induced autophagy was regulated by ROS arising from 5-LOX. Furthermore, silencing expression of 5-LOX using siRNA could also markedly attenuate GA-induced generation of ROS and LC3-II accumulation, demonstrating that 5-LOX is the main source of GA-induced ROS generation (Figure S2A and S2B).

Akt-mTOR signaling has emerged as a key negative regulator of autophagy. Therefore, we examined the phosphorylation status of both Akt and mTOR upon GA treatment. As shown in Figure S3A and S3B, GA treatment significantly inhibited the phosphorylation of both Akt (S473) and mTOR (S2448) in HCT116 cells in both a dose- (Figure S3A) and time- (Figure S3B) dependent manner. p70 S6K, is a downstream target of TORC1 and can serve as a marker of activation of TORC1 [60]. It was therefore of particular interest to determine the phosphorylation status of p70 S6K (S424/T421) following GA treatment. As shown in Figure S3A and S3B, GA treatment resulted in both a dose- and time-dependent dephosphorylation of p70 S6K (S424/T421). To determine whether GA-induced ROS accumulation was involved in regulation of Akt-mTOR signaling, HCT116 cells were treated with GA in the presence or absence of NAC and NDGA (Figure S3C). These results revealed that either NAC or NDGA treatment could markedly attenuate GA-induced dephosphorylation of Akt, mTOR and S6K1. These data showed that GA-induced ROS can inhibit the Akt-mTOR signaling pathway.

### GA Inhibits Colorectal Cancer Growth and Induces Autophagy *in vivo*

**Figure 7:**
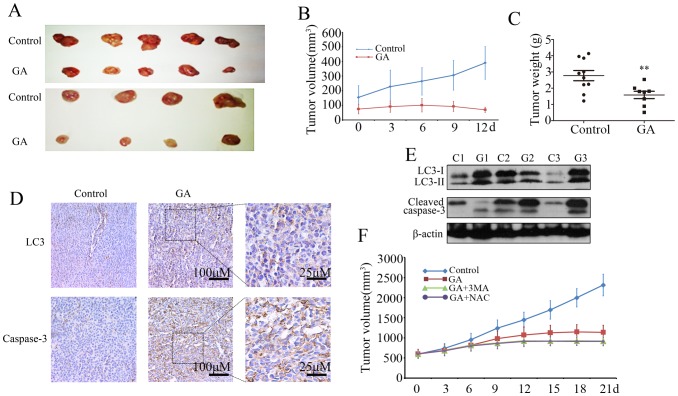
GA inhibits the growth of colorectal cancer and induces autophagy *in vivo*. (**A–E**) C26 tumor-bearing mice were treated with hydroxypropyl-cyclodextrinvehicle (n  = 9) or GA 8 mg/kg (n = 9) twice daily by intraperitoneal injection for 12 days. (A) Excised xenografts showing tumor burden. (B) Mean tumor volume. (C) Tumor weight. (D) Immunohistochemistry analysis of LC3 and cleaved caspase 3 in tumor sections. (E) LC3 and cleaved caspase 3 expression from tumor lysates of three different mice were determined by immunoblot analysis. (**F**) Effect of 3-MA or NAC in combination with GA on growth of C26 tumor xenografts. Growth curve of tumor volumes started from 600 mm^3^.

Finally, the effects of GA on colorectal cancer growth were examined using the C26 colon cancer xenograft model. As shown in Figure 7A–C, GA treatment showed significant decrease of tumor volume and weight compared with the control group. Further, immunohistochemistry analysis of cleaved-caspase 3 accumulation in tumor samples was employed to characterize GA-induced apoptosis *in vivo.* Massive activation of caspase 3 was observed in tumor samples following GA treatment (Figure 7D). To determine whether GA induces autophagy *in vivo*, we examined the expression of LC3 in tumor samples. Tumors from GA-treated mice appeared to have greater levels of LC3 staining compared with tumors from vehicle-treated mice (Figure 7D). Consistent with these results, immunoblot analysis also demonstrated that the levels of LC3-II and activation of caspase 3 in the tumor of GA-treated mice were increased (Figure 7E). Our studies therefore suggest that autophagy may also be involved in GA-induced suppression of tumor growth *in vivo*.

To examine whether ROS-induced autophagy was involved GA-induced suppression of tumor growth *in vivo*, we used the autophagy inhibitor 3-MA or ROS scavenger NAC in combination with GA in a mouse xenograft model. Results suggested that GA in combination with either 3-MA or NAC was clearly more effective in decreasing tumor volume (Fig 7F), indicating that ROS-induced autophagy protected against GA-induced cell death *in vivo.*

## Discussion

A number of recent studies have shown that GA exerts significant anti-proliferative and pro-apoptotic effects on multiple types of human cancer cells *in vitro* and *in vivo* [9], [12], [13], with relative low toxicity and minimal side effects in normal cells [61], [62], [63]. Moreover, GA has been approved for clinical trials in China, although its anticancer mechanisms are not yet fully understood [7], [16]. In the current study, we found that GA could induce caspase 3-dependent apoptosis and inhibit proliferation in colorectal cancer cells, which was coincident with previous reports [40], [63].

Autophagy is one of the main mechanisms responsible for clearing damaged or long-lived proteins and organelles, and is a highly regulated biological process that plays important roles in tissue homeostasis, development and disease [21], [29], [60]. The induction of autophagy by number of therapeutic agents, including GA, has been reported [28], [43], [64]. In this study, we found that GA could enhance autophagic flux in colorectal cancer cells. Autophagy plays a Janus role in cancer treatment [65], [66]. On one hand, when cells are facing metabolic stress autophagy can act as a temporary cell survival pathway by auto-digestion to provide alternative energy or essential building blocks for biosynthesis [66], [67]. On the other hand, autophagy is also known to lead to cell death (type II programmed cell death) [65], [67], [68]. In the current study, we found that autophagy plays a protective role in GA-induced apoptosis of colorectal cancer cells in both *in vitro* and *in vivo* studies, suggesting that autophagy has potential for clinical benefit.

Although autophagy and apoptosis represent distinct cellular processes with often opposing outcomes, they can be induced by the same stimuli, and are extensively interconnected through various crosstalk mechanisms [65], [68], [69]. ROS is one of the typical mediators involved in both apoptosis and autophagy [39], [70]. It is also known that ROS can damage cellular biomacromolecules and even lead to apoptotic cell death. Thus ROS can act as anti-tumorigenic factors and have been observed to be involved in chemotherapy-induced apoptosis in tumor cells [28], [39], [70]. In the present study, 2DE-based proteomics in combination with bioinformatics analyses revealed that 22% of the proteins altered upon GA treatment were involved in redox homeostasis. Recently, GA has been reported to induce ROS accumulation in human hepatoma SMMC-7721 cells, the ovarian cancer cell line (SKOV-3) and multiple myeloma RPMI-8226 cells, contributing to apoptosis by triggering the mitochondrial signaling pathway and activating caspase-3 [40], [41], [42]. However, the role of GA-induced ROS in autophagy has not yet been reported. Growing evidence shows that ROS could monitor autophagy and apoptosis in multiple contexts and cell types and is essential in some cases of drug-induced autophagy and apoptosis such as oxaliplatin [28], [54], [70], [71], [72]. In this study, we found that ROS is required for GA-induced autophagy and against GA-induced apoptosis.

Our proteomics data also indicated that 15% of the altered proteins were involved in lipid metabolism, suggesting that GA treatment may lead to dysregulation of lipid metabolism. In mammalian cells, in addition to mitochondrial respiratory chain malfunction and NOX, ROS are also generated by 5-lipoxygenase (5-LOX) [58], [59]. 5-LOX, a mixed function oxidase, can promote the oxidative metabolism of arachidonic acid (AA) that is released from glycerolphospholipids in the nuclear envelope or the membrane phospholipids, accompanying the synthesis of superoxide anion which will rapidly convert to hydrogen peroxide [58], [59], [73]. In addition, a range of 5-LOX metabolites, such as leukotriene B4 (LTB4), can also induce generation of ROS by stimulating NOX [74], [75]. Our results indicated a major involvement of 5-LOX in the production of ROS upon GA treatment. 5-LOX acts as a downstream mediator in the Rac-signaling pathway leading to the generation of ROS [76]. These ROS could serve as specific second messengers mainly responsible for FAK and subsequent AKT and MAPK (such as ERK, p38) activation. Recently, it was shown that 5-LOX is overexpressed in adenomatous polyps and colon cancer specimens compared with normal colonic mucosa, and 5-LOX expression is closely correlated with tumor size, depth, and vessel invasion [77], [78]. Blockade of 5-LOX or its downstream products (in particular LTB4) reduced colonic cancer cells proliferation both *in vitro* and *in vivo*, indicating that 5-LOX inhibitor may represent a promising candidate chemopreventive agent for colon cancer treatment [79], [80], [81]. In this study, we found that inhibition of 5-LOX suppressed the protective autophagy upon GA-treatment of colon cancer cells, suggesting autophagy was a downstream event of the 5-LOX pathway and may exhibit chemopreventive activities.

The mammalian target of rapamycin (mTOR), has been confirmed as a key negative regulator of autophagy in mammalian cells [60], [82], [83], [84]. In this study, we found that GA suppressed the phosphorylation of Akt, mTOR and p70 S6K (a down regulator of mTOR), suggesting the Akt-mTOR pathway is also involved in GA-induced autophagy in colon cancer cells. In addition, accumulating evidence suggests that 5-LOX and its downstream products (5-HETE) and 5-LOX-induced ROS are also involved in the regulation of PI3K-Akt-mTOR pathways [85], [86], [87], while the enzymes in the PI3K-Akt-mTOR pathways can activate cPLA2, liberating AA and enhancing 5-LOX activity [88]. ROS-induced regulation of AKT-mTOR is mainly through oxidative modification of Cys-dependent phosphatases (i.e., protein tyrosine phosphatases (PTPases) and PTEN) and protein kinases (i.e. PI3K and Akt) [89], [90]. For example, ROS-induced disulfide bond formation between Cys297 and Cys311 can prevent AKT activation [91]. In this study, we found either clearing ROS or inhibition of 5-LOX can attenuate GA-induced inhibition of Akt-mTOR signaling; suggesting Akt-mTOR signaling is associated with GA-induced and ROS-mediated autophagy.

In conclusion, we found that GA could induce autophagy in colorectal cancer cells *in vitro* and *in vivo*, and that inhibition of autophagy augments the anticancer effect of GA, suggesting autophagy plays a protective role in colon cancer cells in this context. These biological effects of GA were tightly regulated by 5-LOX-generated ROS and involved the inhibition of Akt-mTOR pathways. Our study revealed the protective role of ROS-induced autophagy in GA-treated colon cancer cells and suggested potential crosstalk mechanisms between GA-induced autophagy and apoptosis, which will provide new insights into cancer treatment using GA, possibly in combination with autophagy inhibitors.

## Supporting Information

Figure S1 **GA-mediated cell death was caspase dependent in colorectal cancer cells.** HCT116 and SW620 cells were treated GA (1.0 µM for HCT116, 2.0 µM for SW620) in the absence or presence of 20 µM Z-VAD-fmk for 24 h. Cell death was detected by Annexin-V fluorescein isothiocyanate (FITC) and propidium iodide (PI) double staining **(A)**, as well as TUNEL assay **(B)**. * p<0.05; ** p<0.01.
(TIF)

Figure S2 **5-LOX was essential in GA-induced ROS generation and autophagy. (A)** Immunoblot detection of the expression of 5-LOX and LC3 in GA-treated HCT116 cells in the present or absent with siRNA 5-LOX. **(B)** HCT116 cells were treated with Lipofectamine 2000 (Control), 1 µM GA (GA), GA in the presence control siRNA (GA+siControl) or si5-LOX (GA+siRNALOX) for 24 h. And then the intracellular ROS were measured using a Molecular Devices SPECTRAMAX M5 fluorimeter. * p<0.05; ** p<0.01.
(TIF)

Figure S3 **ROS is involved in GA-induced inhibition of Akt-mTOR signaling.** The phosphorylation status of Akt, mTOR and p70 S6K in HCT116 cells treated with indicated concentrations of GA for 24 h **(A)**, with 1 µM of GA for 12 h and 24 h **(B)**, and with1 µM GA in the presence NAC (10 mM) or NDGA for 24 h **(C)** was measured by Western blot analysis. Details of antibodies used are given in Materials and Methods. Actin was used as a loading control.
(TIF)
