# Supplementary figures and images for: ROS-Mediated Autophagy Induced by Dysregulation of Lipid Metabolism Plays a Protective Role in Colorectal Cancer Cells Treated with Gambogic Acid
Source: PLoS One. 2014 May 8;9(5):e96418. doi: 10.1371/journal.pone.0096418 (PMC4014500; doi:10.1371/journal.pone.0096418)

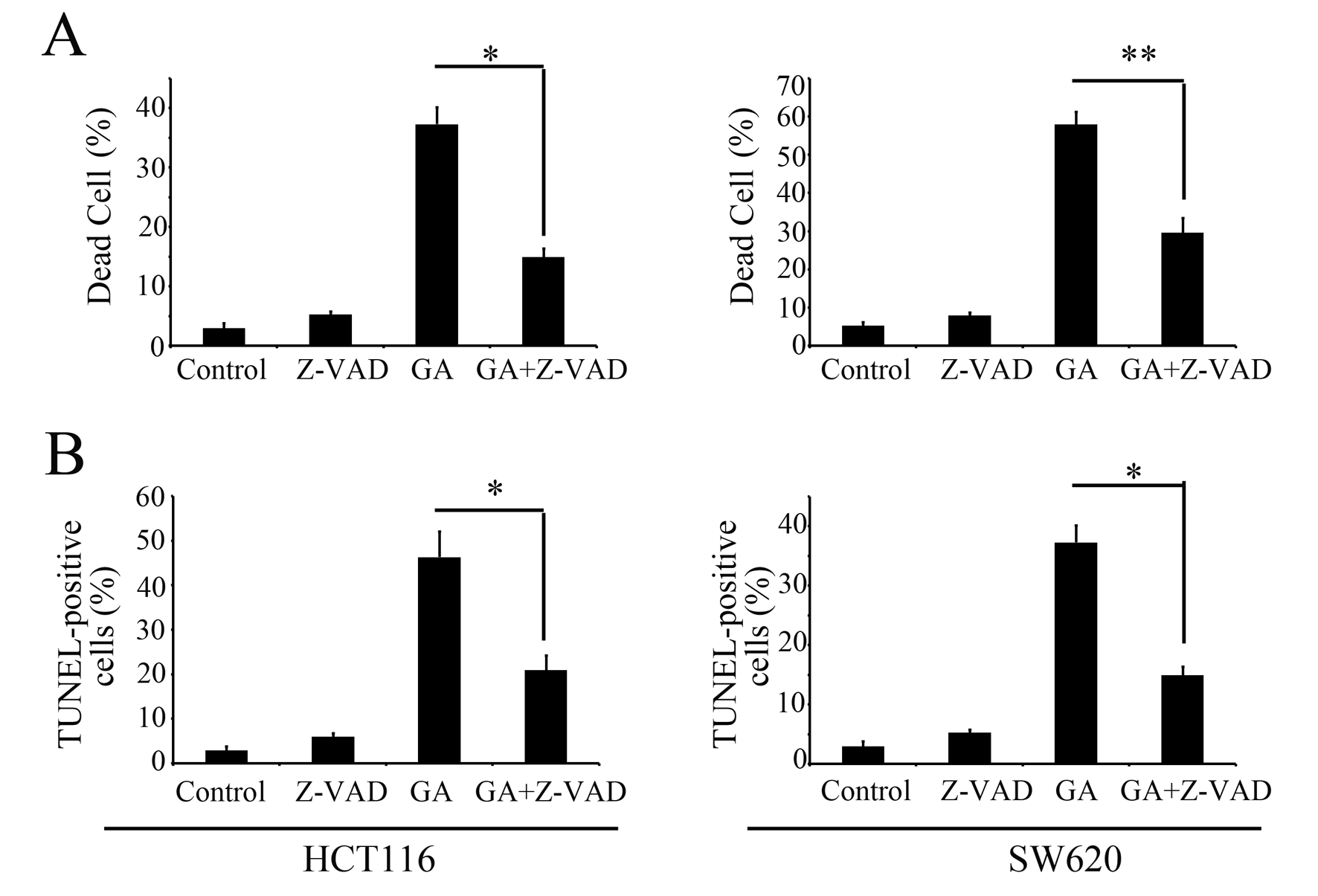

Supplement: Figure S1 — GA-mediated cell death was caspase dependent in colorectal cancer cells. HCT116 and SW620 cells were treated GA (1.0 µM for HCT116, 2.0 µM for SW620) in the absence or presence of 20 µM Z-VAD-fmk for 24 h. Cell death was detected by Annexin-V fluorescein isothiocyanate (FITC) and propidium iodide (PI) double staining (A), as well as TUNEL assay (B). * p<0.05; ** p<0.01. (TIF) [file pone.0096418.s001.tif]

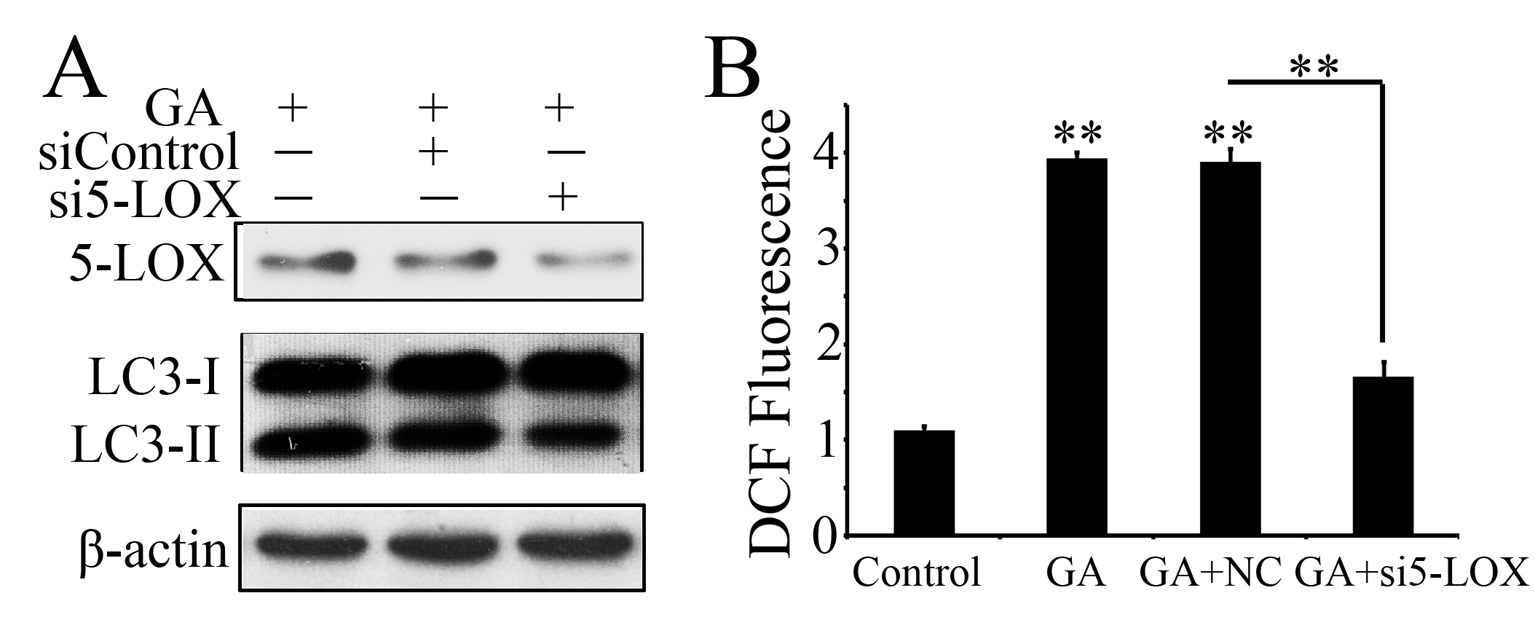

Supplement: Figure S2 — 5-LOX was essential in GA-induced ROS generation and autophagy. (A) Immunoblot detection of the expression of 5-LOX and LC3 in GA-treated HCT116 cells in the present or absent with siRNA 5-LOX. (B) HCT116 cells were treated with Lipofectamine 2000 (Control), 1 µM GA (GA), GA in the presence control siRNA (GA+siControl) or si5-LOX (GA+siRNALOX) for 24 h. And then the intracellular ROS were measured using a Molecular Devices SPECTRAMAX M5 fluorimeter. * p<0.05; ** p<0.01. (TIF) [file pone.0096418.s002.tif]

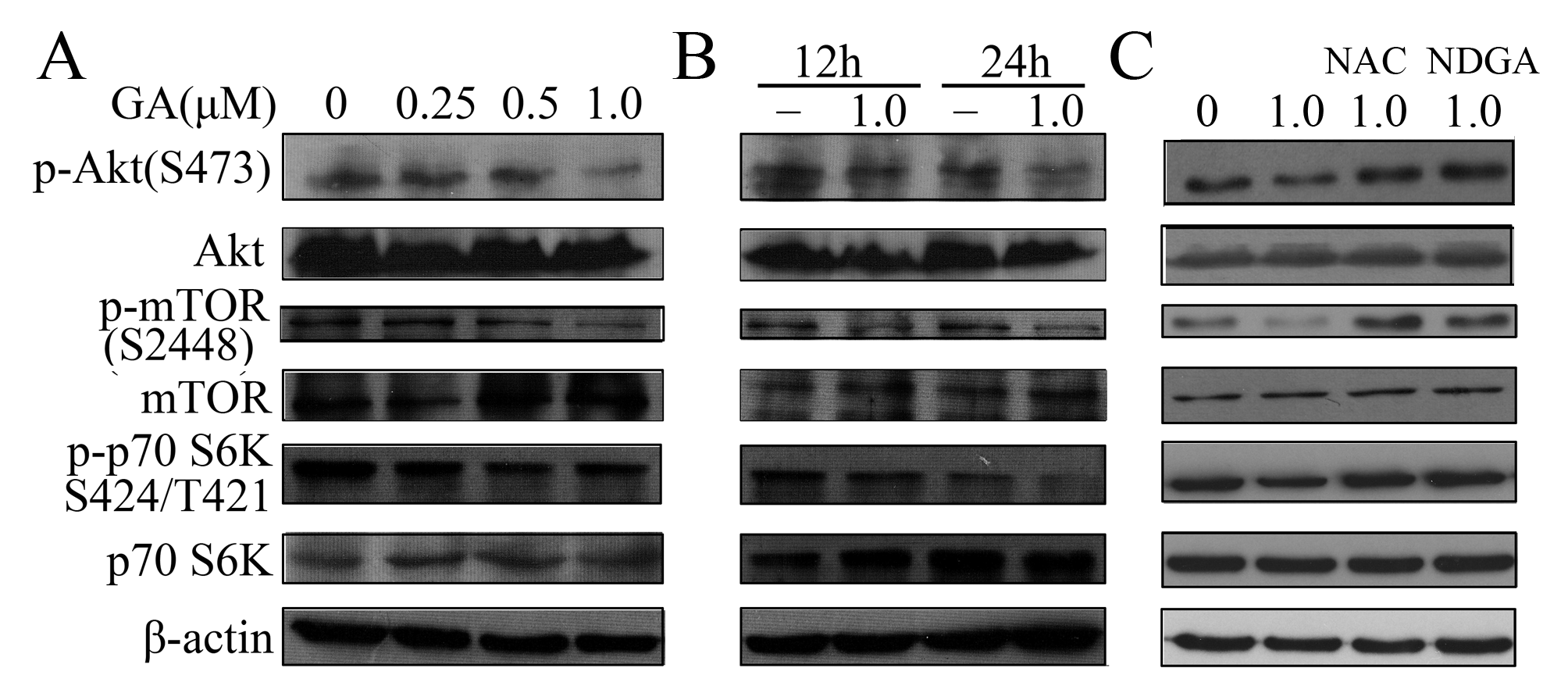

Supplement: Figure S3 — ROS is involved in GA-induced inhibition of Akt-mTOR signaling. The phosphorylation status of Akt, mTOR and p70 S6K in HCT116 cells treated with indicated concentrations of GA for 24 h (A), with 1 µM of GA for 12 h and 24 h (B), and with1 µM GA in the presence NAC (10 mM) or NDGA for 24 h (C) was measured by Western blot analysis. Details of antibodies used are given in Materials and Methods. Actin was used as a loading control. (TIF) [file pone.0096418.s003.tif]
